# A Systematic Review of Cost-Sharing Strategies Used within Publicly-Funded Drug Plans in Member Countries of the Organisation for Economic Co-Operation and Development

**DOI:** 10.1371/journal.pone.0090434

**Published:** 2014-03-11

**Authors:** Lianne Barnieh, Fiona Clement, Anthony Harris, Marja Blom, Cam Donaldson, Scott Klarenbach, Don Husereau, Diane Lorenzetti, Braden Manns

**Affiliations:** 1 Department of Medicine, University of Calgary, Calgary, Alberta, Canada; 2 Department of Community Health Sciences, University of Calgary, Calgary, Alberta, Canada; 3 Centre for Health Economics, Monash University, Melbourne, Australia; 4 Faculty of Pharmacy, University of Helsinki, Helsinki, Finland; 5 Yunus Centre for Social Business & Health, Glasgow Caledonian University, Glasgow, United Kingdom; 6 Department of Medicine, University of Alberta, Edmonton, Alberta, Canada; 7 Department of Epidemiology and Community Medicine, University of Ottawa, Ottawa, Ontario, Canada; Monash University, Australia

## Abstract

**Background:**

Publicly-funded drug plans vary in strategies used and policies employed to reduce continually increasing pharmaceutical expenditures. We systematically reviewed the utilization of cost-sharing strategies and physician-directed prescribing regulations in publicly-funded formularies within member nations of the Organization of Economic Cooperation and Development (OECD).

**Methods & Findings:**

Using the OECD nations as the sampling frame, a search for cost-sharing strategies and physician-directed prescribing regulations was done using published and grey literature. Collected data was verified by a system expert within the prescription drug insurance plan in each country, to ensure the accuracy of key data elements across plans.

Significant variation in the use of cost-sharing mechanisms was seen. Copayments were the most commonly used cost-containment measure, though their use and amount varied for those with certain conditions, most often chronic diseases (in 17 countries), and by socio-economic status (either income or employment status), or with age (in 15 countries). Caps and deductibles were only used by five systems. Drug cost-containment strategies targeting physicians were also identified in 24 countries, including guideline-based prescribing, prescription monitoring and incentive structures.

**Conclusions:**

There was variable use of cost-containment strategies to limit pharmaceutical expenditures in publicly funded formularies within OECD countries. Further research is needed to determine the best approach to constrain costs while maintaining access to pharmaceutical drugs.

## Introduction

Pharmaceutical expenditures account for between 7% and 34% of total health spending across the 34 Organization of Economic Cooperation and Development (OECD) countries [1] and are growing faster than the gross national product in all European countries [2]. Given the increased use of drugs, and as new and more expensive drugs become available, the financial pressure on both individuals and publicly-funded drug plans continues to increase [3]. Publicly-funded drug plans are often included as part of public health insurance as many medications are considered necessary to maintain or improve health, and may not be affordable to many people. However, prescription drug insurance needs to balance the goals of equity and access while acknowledging that budgets are limited [4].

Given differences in socio-demographic characteristics, geo-political systems, and the mandates of the agencies that offer prescription drug insurance, publicly-funded outpatient prescription drug insurance plans have evolved in different ways across different countries leading to variations in the types of citizens covered, cost-sharing strategies used and policies employed to reduce expenditures within publicly funded drug plans. These differences can be thought of as tradeoffs. Higher patient copayments can reduce patient consumption, and possibly encourage patients to use care more efficiently while generating revenue to reduce insurer costs; however, no copayment removes the financial disincentive to forgo care, but risks an inappropriate consumption of pharmaceuticals. Copayments can therefore be viewed as inequitable, as they create a barrier to seek necessary medications to maintain health, particularly in those with a lower socioeconomic status.

Previous reports have noted differences across countries in access to prescription drug insurance, including reimbursement available for selected groups based on age, disease severity or income status [5]–[7], or copayment levels [5], [8], [9]. This study is part of a larger initiative exploring how the different characteristics of publicly-funded prescription drug insurance plans across OECD countries correlate with differences in appropriate access to drugs and drug expenditure, with the goal of informing drug policy. In this article, we report the findings of a systematic review of the utilization of cost-sharing strategies, including the use of copayments, deductibles, premiums, and of physician-directed prescribing regulations in OECD countries. We also highlight unique features of selected systems to showcase some of the innovative approaches to cost sharing along with strategies aimed to reduce inappropriate drug prescribing that could be adapted and adopted elsewhere.

## Methods

### Study sample

The OECD nations formed the sampling frame, as they include both developed and emerging nations from Europe, North America, Latin America and Australasia, representing countries with varied sociocultural characteristics, budgetary restrictions and healthcare systems. Only OECD countries that offered publicly funded health care along with a publicly funded outpatient pharmaceutical insurance system to at least a portion of its citizens were included. Publicly funded drug insurance systems that focused solely on inpatient drugs or drugs for a specific clinical condition, such as cancer, were excluded.

### Database search strategy

Two reviewers searched the following electronic databases, from inception until March 2012: MEDLINE, EMBASE, PubMed. In brief, the key terms included the terms: *drug costs, formularies, prescription drugs, reimbursement mechanisms, and insurance*. The search was developed in consultation with an experienced librarian (Figure S1 for full details). A secondary search was undertaken in the grey literature, focusing on, but not limited to, the various drug reimbursement agency websites. Only the most recent information for each country was retained. Data collection from public documents commenced March 2011 and finished in September 2012.

### Website search strategy

Concurrently with the database searching, we carried out a search of relevant drug agency websites, publicly-funded health insurers, and relevant terms using Google. We searched websites in a systematic manner, first using the site map to identify research and/or publication links and then using the website's search engine to search relevant terms. For all websites, we searched the terms: *reimbursement, pharmaceutical, committee*, and *formulary*. Logs were kept of websites searched, with links to relevant pages saved.

As the majority of the data came from the grey literature, we identified a person familiar with the process through which new prescription drugs are evaluated for inclusion within the formulary within each jurisdiction (“system expert”) and verified the accuracy of subjective data elements abstracted. System experts were identified through peer-reviewed publications, agency websites, or appropriate contacts of the research team. A table of the collected data was e-mailed to the system expert, who was asked to confirm its accuracy or clarify any discrepancies. The experts were invited to enter additional comments and provided information between January and September 2012.

### Variable definition

Recognizing that countries may define cost-sharing strategies differently, we define our terminology as follows:

**Cap.** A limit below which a patient does not pay or has reduced payments for prescriptions. After the cap is reached, payment is required by the patient. All caps are assumed to be annual unless otherwise specified.
**Fixed copayment.** A system where a patient pays a fixed, or set, amount per drug or per prescription.
**Percentage copayment.** A system where a patient pays a set percentage of the amount per drug or per prescription
**Tiered copayments.** A structure where certain drugs (either generic, particularly effective or cost-effective brand name drugs) are assigned a lower copayment (first tier), with non-preferred brand drugs assigned a higher copayment (second tier). A third tier, with an even higher copayment, may be assigned to less preferred brand drugs.
**Deductible.** A limit up to which a patient pays the full cost of the drug. After the deductible is reached, the patient either does not pay or has reduced payments for prescriptions. All deductibles are assumed to be annual unless otherwise specified.
**Premium.** A fixed amount, not related to the number of prescriptions, that a beneficiary must pay to be eligible for prescription drug insurance.
**Maximum out-of-pocket limit.** A limit that is set as a fixed dollar amount or as a percentage of income after which the insurer pays 100% of the drugs. Copayments are in place prior to the limit being reached. All maximum out-of-pocket limits are assumed to be annual unless otherwise specified.

### Data abstraction

Data was gathered on the characteristics of healthcare systems, the details relating to outpatient pharmaceutical insurance within each country, and the specifics of the pharmaceutical reimbursement decision-making systems. All data were extracted in duplicate by two members of the research team (LB and FC) with disagreements resolved by consensus. A kappa statistic for agreement between reviewers was calculated for each country, and agreement was between 0.8 and 1.0, for all data collection elements. Country experts were identified through peer-reviewed publications, agency websites, or appropriate contacts of the research team. A table of the collected data was e-mailed to the country expert, who was asked to confirm its accuracy or clarify any discrepancies. The experts were invited to enter additional comments. A list of the “country experts” is available upon request.

### Data synthesis

A description of the data collected on use and structure of cost-sharing strategies (copayments, caps and deductibles) (as defined above), as well as physician-directed prescribing regulations (compulsory or non-compulsory prescription guidelines, monitoring and comparison of prescription patterns and volumes, incentives and sanctions) are presented separately for each country. In addition to a tabular summary, a qualitative summary is given of three unique strategies directed at physician prescribing behavior.

## Results

The search yielded 2,466 citations, 41 of which were selected for full-text review. Of these, 5 studies, met our inclusion criteria of addressing cost-sharing mechanisms in publicly-funded health care systems. Through searching the grey literature, 98 reports were identified along with prescription drug insurance websites (Table S1)

### Availability of publicly funded prescription drug insurance

Of the 34 OECD countries, one country (Chile) was excluded as there is no publicly-funded prescription drug insurance provided in the country. Given the unique characteristics of health care systems in Ireland, Mexico and the U.S., data were collected for select national publicly-funded insurance plans: General Medical Services Scheme in Ireland for those over 70 and those with lower income; Seguro Popular in Mexico for those with low income; and Part D Medicare in the U.S, for those over the age of 65 as they cover a large proportion of the population. As Canada and Israel have several drug insurance plans, none of which are available at a national level to all citizens, we provide an overview of system characteristics. Further, we included both England and Scotland from the United Kingdom as the characteristics of prescription drug insurance varies between the two systems. Thus, 34 systems were included in the final analysis: Australia, Austria, Belgium, Canada, Czech Republic, Denmark, England, Estonia, Finland, France, Germany, Greece, Hungary, Iceland, Ireland- General Medical Services Scheme, Israel, Italy, Japan, Luxembourg, Mexico- Seguro Popular, Netherlands, New Zealand, Norway, Poland, Portugal, Scotland, Slovakia, Slovenia, South Korea, Spain, Sweden, Switzerland, Turkey, and United States (US)- Part D Medicare.

Five countries included in this review do not provide universal prescription drug insurance for all citizens, with or without a premium: Canada, Estonia, Israel, Mexico and the United States. Prescription drug insurance in Estonia varies by age and employment status; however, over 95% of the population is covered by prescription drug insurance. Seguro Popular in Mexico covers those who are unable to get private insurance due to lower income and is provided with no premiums by the government. Medicare part D in the United States is available to all US citizens aged 65 or over. Those on disability or with end-stage renal disease on dialysis under the age of 65 are also eligible. In Canada and Israel, plans vary by region of the country or by health fund, respectively, and may be subject to a premium.

### Cost sharing policies within publicly funded prescription drug insurance systems

The use of cost-sharing mechanisms varied significantly (Table I). Seventeen countries (Belgium, Czech Republic, Denmark, England, Estonia, Greece, Finland, Hungary, Italy, Luxembourg, New Zealand, Norway, Poland, Slovenia, South Korea, Spain, and Turkey) had reduced or no copayments for those with certain conditions, most often chronic diseases, though the type of chronic conditions varied from system to system. Five countries had copayments which varied depending on the type of drug or its indication for use. In Portugal, the copayment for drugs was dependent on the deemed essential nature of the pharmaceutical or class of medications, while in Greece and Sweden, there are no copayments explicitly for insulin. In Iceland and Slovakia, all pharmaceuticals deemed vital by the agency are reimbursed in full. Finally, copayments varied by socio-economic status, either income or employment status, or with age in 15 countries: Australia, Belgium, Czech Republic, England, Estonia, Greece, Hungary, Italy, Japan, New Zealand, Norway, Slovenia, South Korea, Spain, and Turkey. Copayments varied as either fixed, percentage, or a combination of both, with some systems placing a maximum dollar value on the percentage. In the Netherlands, the copayment was the difference between the retail price and the reference price, set by the agency. Thirteen countries employed a maximum out-of-pocket limit for the beneficiary. These limits were either fixed (Australia, Finland, Japan, Norway, South Korea), varied by the annual income of the patient (Austria, Germany, Luxembourg), age (Czech Republic, Switzerland), or presence of chronic conditions (Belgium, Denmark, Germany). Though there was no set maximum out-of-pocket limit for the Medicare system in the US, depending on the specifics of the plan, the copayment reduces to less than 5% once a threshold is reached.

**Table 1:** Use of cost-sharing and cost-containment policies within included OECD countries.

	Copayments								Sources*
Country	Use of copayment	Vary by condition	Vary by type of drug	Vary by socio-economic state	Fixed or percentage	Maximum out –of-pocket limit (MOPL)	Cap	Deductible	
Australia	Yes	No	No	Yes	Fixed	Fixed, dependent on type of patient	No	No	[9], [34]–[37]
Austria	Yes	No	No	No	Fixed	2% of annual income	No	No	[10], [38]–[40]
Belgium	Yes	Yes	No	Yes	Percentage	Dependent on type of patient	No	No	[41]–[45]
Canada	Varies by plan	No	No	Varies by plan	Varies by plan	Varies by plan	Varies by plan	Varies by plan	[7], [46]–[51]
Czech Republic	Yes	Yes	No	Yes	Fixed	Set at 200€; for children under 18 and adults over 65, set at 100€	No	No	[52], [53]
Denmark	Yes	Yes	No	No	Both	Set at 406€ for chronically ill patients	No	Yes	[54], [55]
England	Yes	Yes	No	Yes	Fixed	No	No	No	[45], [56]–[59]
Estonia	Yes	Yes	No	Yes	Both	No	No	No	[60], [61]
Finland	Yes	Yes	No	No	Percentage	Set at 672€; subsequent costs are reimbursed in full after a fixed 1.50€ copayment	No	No	[62]–[64]
France	Yes	No	No	No	Both	No	No	No	[45], [65]–[67]
Germany	Yes	No	No	No	Both	Set at 2% of net income; 1% of net income for chronically ill patients	No	No	[45], [68]–[72]
Greece	Yes	Yes	Yes	Yes	Percentage	No	No	No	[73], [74]
Hungary	Yes	Yes	No	Yes	Percentage	No	No	No	[75], [76]
Iceland	Yes	No	Yes	No	Percentage	No	No	No	[77]
Ireland**	Yes	No	No	No	Fixed	19.50€ per month per family	No	No	[78]–[83]
Israel	Varies by plan	Varies by plan	Varies by plan	Varies by plan	Varies by plan	Varies by plan	No	No	[84], [85]
Italy	Yes	Yes	No	Yes	Fixed	No	No	No	[45], [86]–[88]
Japan	Yes	No	No	Yes	Percentage	Set at 80,000 yen monthly	No	No	[89]–[93]
Luxembourg	Yes	Yes	No	No	Percentage	2.5% of net income	No	No	[94], [95]
Mexico+	No					No	No	No	[96], [97]
Netherlands	Yes	No	No	No	Difference between reference price and retail	No	No	Yes	[45], [98]
New Zealand	Yes	Yes	No	Yes	Fixed	No	No	No	[35], [99]–[101]
Norway	Yes	Yes	No	Yes	Both	Set at 216€ and 63€ per prescription	No	No	[102]
Poland	Yes	Yes	No	No	Both	No	No	No	[103]–[105]
Portugal	Yes	No	Yes	No	Percentage	No	No	No	[106], [107]
Scotland	No						No	No	[57]
Slovakia	Yes	No	Yes	No	Both	No	No	No	[108], [109]
Slovenia	Yes	Yes	No	Yes	Percentage	No	No	No	[105], [110]
South Korea	Yes	Yes	No	Yes	Percentage	Set at 2, 3 or 4 million KRW depending on health insurance plan	No	No	[93], [111]–[116]
Spain	Yes	Yes	No	Yes	Percentage	No	No	No	[45], [117], [118]
Sweden	Yes	No	Yes	No	Percentage	No	No	Yes	[22], [119]–[123]
Switzerland	Yes	No	No	No	Percentage	Set at 700 CHF for adults and 350 CHF for children	Yes	Yes	[124], [125]
Turkey	Yes	Yes	No	Yes	Percentage	No	No	No	[96], [105], [126]
US++	Varies	No	No	No		Copayment reduces to 5% after limit	Varies by plan. Step therapy, prior authorization and cost tiers	[127]	

* in addition to system experts and agency websites.

** General Medical Services Scheme.

+ Seguro Popular plan.

++ Medicare.

### Patient and system-level restrictions

Throughout the included OECD countries, only one system employed a premium as a mechanism to fund the prescription drug insurance plan: Medicare part D, where premiums vary according to plan and income. Only one country employed a system of caps; Switzerland allowed reduced copayments for brand name drugs, up to a cap of 933 CHF, after which patients were responsible to pay for 100% of the prescription. This cap did not apply to generic drugs. Four systems in the OECD used a deductible: Denmark, the Netherlands, Sweden, and Medicare part D. In the Netherlands, there is no copayment or reimbursement under 170 euros of annual expenses. In Denmark and Sweden, the percentage copayment decreases throughout the year based on consumption. The amount of the deductible for Medicare part D varies according to plan and ranges from no deductible to 325 USD.

### Volume control measures aimed at physicians

Cost-containment strategies targeting physician prescribing were seen in several jurisdictions. Sixteen countries enforced guideline-based prescribing, either compulsory or non-compulsory (Table II). Physician prescription patterns and volume were monitored in nineteen countries, and in several of these countries, the patterns and volume of physician prescribing were benchmarked against others (Austria, Belgium, Denmark, England, Estonia, Finland, Hungary, and Slovenia). Incentive structures in the form of rewards were used in four countries (Austria, Belgium, England, and Spain) and sanctions for over-prescribing were seen in three countries (Austria, Belgium, and Luxembourg).

**Table 2:** Strategies to increase appropriate prescribing.

Country	Compulsory prescription guidelines*	Non-compulsory prescription guidelines	Prescription pattern and volume monitored	Prescription pattern and volume compared to others	Incentives	Sanctions	Other
Australia	X	X	X	X	X	X	
Austria	X	X	✓	✓	✓	✓	
Belgium	X	✓	✓	✓	✓	✓	
Canada	X	X	X	X	X	X	No regulation of prescribing
Czech Republic	X	X	X	X	X	X	Only specialists can prescribe new and more expensive pharmaceuticals
Denmark	X	✓	✓	✓	X	X	Interactive database to facilitate self-monitoring
England	X	✓	✓	✓	✓	X	
Estonia	✓	X	✓	✓	X	X	Only one pharmaceutical per prescription
Finland	X	✓	✓	✓	X	X	Rational prescribing program for doctors
France	✓	X	✓	X	X	X	
Germany	✓	X	X	X	X	X	
Greece	X	X	X	X	X	X	
Hungary	X	X	✓	✓	X	X	
Iceland	X	X	X	X	X	X	
Ireland	X	✓	X	X	X	X	Certain insurance schemes have the right to influence the prescribing of doctors
Israel	X	X	✓	X	X	X	
Italy	✓	X	X	X	X	X	
Japan	X	X	✓	X	X	X	
Luxembourg	X	✓	✓	X	X	✓	Sanctions are rarely applied
Mexico	X	X	X	X	X	X	
Netherlands	X	✓	✓	X	X	X	
New Zealand	X	X	X	X	X	X	
Norway	X	X	✓	X	X	X	
Poland	X	X	X	X	X	X	
Portugal	X	✓	X	X	X	X	
Scotland	X	✓	✓	X	X	X	
Slovakia	✓	X	✓	X	X	X	Insurance companies monitor the ratio of prescribed originals versus generics for contract doctors
Slovenia	X	X	✓	✓	X	X	Only one pharmaceutical per prescription, for a one-month supply
South Korea	X	X	✓	X	X	X	
Spain	X	X	✓	X	✓	X	Bonuses to physicians if pharmaceutical expenditure does not exceed forecasted growth at the regional level
Sweden	X	✓	✓	X	X	X	County councils are responsible for prescribing policies in their respective region
Switzerland	X	X	X	X	X	X	No regulation of prescribing
Turkey	✓	X	X	X	X	X	Guidelines for number of items, dose and treatment time as well
US Medicare	X	X	X	X	X	X	Step therapy and prior authorization used

✓ = yes; X = no.

* may not be available for all conditions.

### Strategies to change physician-prescribing practice

Though strategies to change physician-prescribing practice were seen in several countries, the following are three unique system cases that utilize several physician-directed strategies aimed at reducing costs through rational prescribing. In Austria, physician's prescription patterns are monitored, and volume is benchmarked against others, to ensure that the most cost-effective drug is chosen when a physician has several similar therapeutic options. In the case of serious discrepancies, doctors may be forced to pay back the difference between the price of the prescribed product and the average prescribed price [10]. This is very rare and is usually solved through arbitration. In Belgium, consensus meetings are held at least twice a year to formulate recommendations for all prescribing physicians. Agreements exist between some insurers and doctors that allow a bonus to be paid to the doctor if prescriptions for antibiotics, for example, fall below a certain threshold. Doctors are obliged to prescribe a certain amount of lower priced pharmaceuticals, defined as prescribing by active ingredient; by generic brand name; or prescribing originators priced at the reference price level. If a doctor fails to attain the national minimum of these lower priced pharmaceuticals, which was set at 27% of all prescriptions in 2008, prescriptions are monitored for a further six months. If no improvement is seen by the end of this period, the doctor is liable for a fine of between €1,000 and €5,000. This has doubled the number of lower priced pharmaceuticals prescribed, from 2004 to 2005 [11]. Finland uses a computer program for rational prescribing, informed by the information on effectiveness and cost-effectiveness submitted by manufacturers to the drug reimbursement agency, facilitates physicians choosing the recommended drug based on efficiency, safety, and patient appropriateness. Each year, doctors receive a summary of their prescriptions and costs from the prescribing system, including data on the number of prescriptions, distribution of patients' age and gender, and average cost per prescription, to other doctors in the same region. The Finnish Medical Society has issued national clinical treatment guidelines for common diseases, with the most recent edition including economic information.

## Discussion

Publicly-funded prescription drug plans are constantly evolving. The majority of countries included in this review, however, utilized some form of cost-sharing strategy, most commonly copayments, though the magnitude of copayment varied across countries, and within countries by socioeconomic class, age or presence of chronic condition. In addition, we noted that several countries used unique cost-containment features including varying levels of copayment for generic versus brand name drugs and a copayment that is calculated based on the difference between the retail price and the reference price. Strategies aimed at encouraging appropriate prescribing by physicians were noted in several countries, in various forms, as an effort to reduce volume and/or expenditures.

The issue of cost constraint within health systems has become more relevant since the recent 2008 financial crisis. Indeed, during the recent financial crisis, a study noted that 89 pharmaceutical policy changes across 23 countries, many of which were OECD, were implemented [12]. Though these changes may not all be directly related to the financial crisis, it highlights the desire of publicly-funded drug plans to shift the burden of increasing expenditures onto the patient. Our review suggests that countries have different approaches to limiting expenditures for their publicly-funded drug plans and place different priorities on who should be able to access prescription drug insurance, and at what cost. These decisions, as expected, have impacts on equity in any given system. The relative value of each system rests on the principles of each society and needs to be evaluated in the context of the trade-offs that copayments offer.

Copayments provide significant opportunities in a prescription drug insurance plan to maximize their budget: one study found that doubling a patient's co-payment in a given plan, regardless of the type of co-payment, reduces average annual drug spending by one-third [13]. Increasing co-payments, however, has been shown to decrease drug usage in an effort by the patient to maintain their overall costs; of concern, patients are unlikely to reduce consumption of only less effective medications. One study found that for every $10 increase in co-payments, average compliance fell by 5 percentage points and that lower compliance resulted in greater use of other more expensive medical services [14]. The impact on clinical outcomes of potential decreased drug use has been examined previously, though the evidence is conflicting. The RAND Health Insurance Experiment examined the effect of copayments on health outcomes and while they noted no evidence that copayments affected clinical outcomes for patients overall, they noted that in people with lower incomes the presence of a copayment was a barrier to seeking care [15]. The RAND study was conducted in the early 1980s, however, before medications became a cornerstone of treating many chronic conditions. Since then, studies have noted similar findings for the elderly, and low-income individuals, where the introduction of a cost-sharing policy decreased the use of essential drugs and increased the incidence of serious adverse events [16]

Some systems attempted to mitigate the potential clinical impact of copayments by differentially lowering co-payments for patients with specific chronic conditions or for certain medication classes. This strategy has little evidence to support or refute its utility. One study showed that in chronically ill patients, doubling co-payments from $5 to $10 caused greater delays in starting treatment [17]. Another study noted that in a high risk group of US Veteran's Administration patients with coronary heart disease, increasing copayment by $5 per prescription resulted in a 30–40% lower adjusted odds of adherence, across a variety of measures, for patients who were subject to copay [18]. Finally, other studies have noted that lower copayments have also been noted to improve adherence while not affecting overall expenditure in people with chronic conditions [19]–[25].

Placing a cap on the amount of benefit a patient can receive during a given time period was another cost-sharing strategy, though this was only used by one country (Switzerland) and in certain plans in Canada and the United States. Though the evidence is limited, one study found that among the chronically ill, patients who had reached their benefit cap are more likely to stop taking their medications than those who haven't [26]. Further, of those who stopped their medications, only a minority resumed therapy in the first three months after their coverage returned. The impact of this on clinical outcomes and overall costs is uncertain [26].

Targeting the prescribing practices of physicians is another strategy that health care payers have used to reduce excessive prescribing. Since decisions about which medications are needed for which conditions are generally made by physicians, it might be argued that a higher burden for reducing expenditures should be placed on the healthcare provider through the use of incentives [27]. We noted several strategies aimed at physician prescribing across different OECD countries in an effort to engage physicians and transfer some of the responsibility for cost-containment measures. France implemented mandatory practice guidelines, including prescribing, in 1994 [28]. Though the sanctions have since been removed since implementation, the implementation of the guidelines did result in an overall net reduction in drug expenditure. However, because of the lack of sanctions imposed on physicians, compliance is low, and the effectiveness of this policy is uncertain [29]. In 2004, the Norwegian Medicines Agency made thiazides the only reimbursed drug class for uncomplicated hypertension, in an effort to reduce expenditures [30]. The introduction of the new rule significantly changed prescription practices, with no change in clinical outcomes. However, the expected decrease in drug expenditures was not observed. In 2009, Finland began to implement a system that provides guidelines (which consider both effectiveness and cost-effectiveness) electronically to physicians to optimize use of medications [31]. However, no evaluation on the efficacy of these electronic guidelines or the strategies seen in other countries has been conducted. A careful evaluation of these strategies, and their impact on clinical outcomes, is needed to inform health systems about the impact of such strategies.

We were unable to infer the relative benefits of the above cost-sharing strategies on publicly-funded prescription drug expenditure plans, as to the best of our knowledge, limited observational evidence exists within these systems regarding the impact of cost-sharing strategies on global system expenditures [32]. In dynamic systems that are not immune to changes in the social, demographic and economic climate, it is difficult to assess and extrapolate the relative impact of each cost-containment measure on pharmaceutical expenditures. Though, as identified above, there are isolated reports on the impact of copayments on individual outcomes (effectiveness, adherence, and patient expenditure) [13], [15]–[25], [33], studies examining the impact of these different measures at a national plan level are lacking.

Our study has limitations. The majority of the literature identified in the systematic review came from published and grey literature sources. Given the dynamic and responsive nature of the drug reimbursement systems, this may not capture the current state of the systems. In addition, obtaining information from experts involved in the drug plan decision-making process itself may have resulted in biased information. However, we mitigated this bias by accessing several sources of information and targeting individuals within the processes who are likely to be objective (chairs of the committees and academics publishing in this area). Future work should focus on evaluating the ability of the implemented tools to contain costs while optimizing clinical benefits.

Prescription-drug insurance plans are one of the most powerful policy levels available for both controlling expenditures in a health system and affective compliance and management among patients. Limiting pharmaceutical expenditures is a concern for many countries across various geographic and health systems. There are similarities and differences in the use of cost-containment measures and strategies aimed at reducing physician prescribing practices across the OECD countries. However, the potential impact of these measures on pharmaceutical expenditures is unknown at the health system level, despite evidence indicating that increasing copayments for patients may negatively affect clinical outcomes for chronic conditions. Further research is needed to determine the best approach to constrain costs, while maintaining access to pharmaceutical drugs.

## Supporting Information

Checklist S1 PRISMA Checklist.
(DOC)

Diagram S1 PRISMA Flow Diagram.
(DOC)

Figure S1 Draft Search Strategy for MEDLINE.
(PDF)

Table S1 Drug reimbursement agencies and data sources used.
(PDF)
