# Supplementary material for: A Systematic Review of Cost-Sharing Strategies Used within Publicly-Funded Drug Plans in Member Countries of the Organisation for Economic Co-Operation and Development
Source: PLoS One. 2014 Mar 11;9(3):e90434. doi: 10.1371/journal.pone.0090434 (PMC3949707; doi:10.1371/journal.pone.0090434)
Supplement: Diagram S1 — PRISMA Flow Diagram. (DOC) [file pone.0090434.s002.doc]

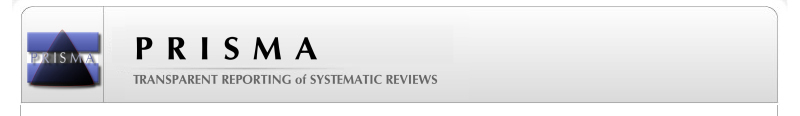
**PRISMA 2009 Flow Diagram**

**Screening**

**Included**

**Eligibility**

**Identification**

Records identified through database searching
(n = 3,237 )

Additional records identified through other sources
(n = 101 )

Records after duplicates removed
(n = 2,567)

Records screened
(n = 2,567 )

Records excluded
(n = 2,425 )

Full-text articles assessed for eligibility
(n = 142 )

Full-text articles excluded, with reasons
(n = 36)

Not relevant (n=36)

Studies included in qualitative synthesis
(n = 106 )
