# Supplementary material for: A Systematic Review of Cost-Sharing Strategies Used within Publicly-Funded Drug Plans in Member Countries of the Organisation for Economic Co-Operation and Development
Source: PLoS One. 2014 Mar 11;9(3):e90434. doi: 10.1371/journal.pone.0090434 (PMC3949707; doi:10.1371/journal.pone.0090434)
Supplement: Figure S1 — Draft Search Strategy for MEDLINE. (PDF) [file pone.0090434.s003.pdf]

Draft Search Strategy for MEDLINE

1. *Drug Costs/
2. *economics, pharmaceutical/
3. *drug therapy/ec
4. *formularies as topic/
5. *Drug Prescriptions/ec, lj [Economics, Legislation & Jurisprudence]
6. *Prescription Drugs/
7. *Economics, Pharmaceutical/
8. *Pharmaceutical Preparations/ec, sd [Economics, Supply & Distribution]
9. *Technology Assessment, Biomedical/
10. 1 or 2 or 3 or 4 or 5 or 6 or 7 or 8 or 9
11. exp National Health Programs/ or exp regional health planning/ or state government/ or state medicine/ or financing, government/ or government programs/ or universal coverage/
12. exp Medicare/
13. exp Medicaid/ or *policy making/ or *health policy/
14. exp *Reimbursement Mechanisms/
15. 11 or 12 or 13 or 14
16. 10 and 15
17. 11 or 12 or 13
18. *Insurance, Pharmaceutical Services/ec, lj, og [Economics, Legislation & Jurisprudence, Organization & Administration]
19. 17 and 18
20. Technology Assessment, Biomedical/ and reimbursement mechanisms/
21. ((public* or national or government* or federal*) adj5 (drug or drugs or medication* or pharmaceutical*) adj5 (plan or plans or program* or mechanism* or reimburs* or funded or funding or coverage)).tw.
22. 16 or 19 or 20 or 21
23. limit 22 to english language
24. exp australia/ or exp cities/
25. exp Austria/
26. Belgium/
27. exp Canada/
28. exp Chile/
29. north america/ or exp canada/ or mexico/ or exp united states/ or israel/ or turkey/ or exp japan/ or exp korea/ or exp australia/ or europe/ or estonia/ or czech republic/ or hungary/ or poland/ or slovakia/ or slovak republic/ or slovenia/ or finland/ or exp france/ or exp germany/ or exp great britain/ or greece/ or iceland/ or ireland/ or exp italy/ or luxembourg/ or netherlands/ or portugal/ or denmark/ or norway/ or sweden/ or spain/ or switzerland/
30. New Zealand/
31. (OECD or european union or commonwealth or north america* or united kingdom).tw.
32. (Australia or Austria or Belgium or Canada or Chile or Czech Republic or Denmark or Europ* or Estonia or Finland or France or Germany or Greece or Hungary or Iceland or Ireland or Israel or Italy or Japan or Korea or Luxembourg or Mexico or North America* or Netherlands or New Zealand or Norway or OECD or Poland or Portugal or Slovak Republic or Slovenia or Spain or Sweden or Switzerland or Turkey or United States or United Kingdom).tw.
33. 24 or 25 or 26 or 27 or 28 or 29 or 30 or 31 or 32
34. 23 and 33
35. limit 34 to (case reports or comment or editorial or letter)
36. 34 not 35
37. limit 36 to clinical trial, all
38. 36 not 37

Note: Search terms for other databases, while based on the MEDLINE template above, were revised as needed to take advantage of the unique features and indexing capabilities of each resource
