# Supplementary material for: A Systematic Review of Cost-Sharing Strategies Used within Publicly-Funded Drug Plans in Member Countries of the Organisation for Economic Co-Operation and Development
Source: PLoS One. 2014 Mar 11;9(3):e90434. doi: 10.1371/journal.pone.0090434 (PMC3949707; doi:10.1371/journal.pone.0090434)
Supplement: Table S1 — Drug reimbursement agencies and data sources used. (PDF) [file pone.0090434.s004.pdf]

Drug reimbursement agencies and data sources used

Country	Agency name	Website	Data sources
**Australia**	Pharmaceutical Benefits Scheme	www.pbs.gov.au/pbs/home	(1-5)
**Austria**	Federation of Austrian Social Insurance Institutions	www.hauptverband.at	(6-9)
**Belgium**	National Institute for Health and Disability Insurance	www.riziv.be	(10-14)
**Canada**	Common Drug Review	www.cadth.ca/index.php/en/cdr	(15-21)
**Czech Republic**	State Institute for Drug Control	www.sukl.cz	(22, 23)
**Denmark**	Danish Medicines Agency	www.dkma.dk	(24, 25)
**England**	National Institute for Health and Clinical Excellence	www.nice.org.uk/	(14, 26-29)
**Estonia**	Ministry of Social Affairs, with advisement from Estonian Health Insurance Fund and Estonian State Agency of Medicines	www.sm.ee	(30, 31)
**Finland**	Pharmaceuticals Pricing Board	www.hila.fi	(32-34)
**France**	Haute Autorité de Santé	www.has-sante.fr	(14, 35-37)
**Germany**	Federal Joint Committee	www.g-ba.de/	(14, 38-42)
**Greece**	Ministry of Health and Social Solidarity	www.yyka.gov.gr/	(43, 44)
**Hungary**	Department of Reimbursement of the National Health Insurance Fund Administration	www.oep.hu	(45, 46)
**Iceland**	Icelandic Medicines Agency	www.lyfjastofnun.is/	(47)
**Ireland**	Health Service Executive	www.hse.ie	(48-53)
**Israel**	Medical Technology Administration	www.health.gov.il	(54, 55)
**Italy**	Italian Medicines Agency	www.agenziafarmaco.it	(14, 56-58)
**Japan**	Ministry of Health, Labour and Welfare	www.mhlw.go.jp/	(59-63)
**Luxembourg**	National Health Fund	www.cns.lu	(64, 65)
**Mexico**	Inter-institutional Formulary Board	N/A	(66, 67)
**Netherlands**	Ministry of Health, Welfare and Sport	www.rijksoverheid.nl/	(14, 68)
**New Zealand**	Pharmaceutical Management Agency	www.pharmac.govt.nz/	(3, 69-71)
**Norway**	Norwegian Medicines Agency	www.legemiddelverket.no	(72)
**Poland**	Ministry of Health	www.mz.gov.pl/	(73-75)
**Portugal**	National Authority of Medicines and Health Products	www.infarmed.pt	(76, 77)
**Scotland**	Scottish Medicines Consortium	www.scottishmedicines.org.uk	(27)
**Slovakia**	Ministry of Health	www.health.gov.sk	(78, 79)
**Slovenia**	Health Insurance Institute	www.zzzs.si	(75, 80)
**South Korea**	Health Insurance Review Agency	www.hira.or.kr/	(63, 81-86)
**Spain**	Ministry of Health	www.msps.es/	(14, 87, 88)
**Sweden**	Dental and Pharmaceutical Benefits Agency	www.lakemedelsformansnamnden.se/	(89-94)
**Switzerland**	Federal Office of Public Health	www.bag.admin.ch	(95, 96)
**Turkey**	Social Security Institutes	www.sgk.gov.tr	(66, 75, 97)
**United States – Medicare**	Centers for Medicare and Medicaid Services	www.cms.gov/	(98)

1. Department of Health and Aged Care. The Australian Health Care System: An Outline. 2000.
2. Yoongthong W, Hu S, Whitty J, Wibulpolprasert S, Sukantho K, Thienthawee W, et al. National drug policies to local formulary decisions in Thailand, China, and Australia: drug listing changes and opportunities. Value in Health. 2012;15:S126-31.
3. Streat S, Munn S. Health economics and health technology assessment: Perspectives from Australia and New Zealand. Crit Care Clin. 2012;28:125-33.
4. Lopert R. Evidence-based decision-making within Australia's pharmaceutical benefits scheme. Issue Brief (Commonw Fund). 2009;60:1-13.
5. Department of Health and Ageing. Guidelines for preparing submissions to the Pharmaceutical Benefits Advisory Committee (Version 4.3). Canberra2008.
6. Wild C. Austria: History of health techcnology assessment during the past 20 years. International Journal of Technology Assessment in Heatlh Care. 2009;25:74-81.
7. Godman B, Bucsics A, Burkhardt T, Haycox A, Seyfriend H, Weininger P. Insight into recent reforms and initiatives in Austria: Implications for key stakeholders. Expert Review of Pharmacoeconomics and Outcomes Research. 2008;8:357-71.
8. Walter E, Zehetmayr S. Guidelines on Health Economic Evaluaion: Consensus paper. Vienna: Institut fur Pharmaokonomische Forschung, 2006.
9. Leopold C, Habl C. Pharmaceutical Pricing and Reimbursement Information: Austria. Vienna: Gesundheit Osterreich GmbH / Geschaftsbereich OBIG (GOG/OBIG), 2008.
10. Gerkens S, Merkur S. Belgium: Health System Review. European Observatory on Health Systems and Policies, 2010.
11. Cleemput I, van Wilder P, Huybrechts M, Vrijens F. Belgian methodological guidelines for pharmacoeconomic evaluations: toward standardization of drug reimbursement requests. Value in Health. 2009;12:441-9.
12. Cleemput I, Crott R, Vrijens F, Huybrechts M, van Wilder P, Ramaekers D. Guidelines for pharmaco-economic evaluations in Beligum Brussels: Centre federal d'expertise des soins de sante, 2006.
13. DeSwaef A, Antonissen Y. Pharmaceutical Pricing and Reimbursement Information: Belgium. Vienna: Gesundheit Osterreich GmbH / Geschaftsbereich OBIG (GOG/OBIG), 2008.
14. Garattini L, Cornago D, De Compadri P. Pricing and reimbursement of in-patent drugs in seven European counries: A comparative analysis. Health Policy. 2007;82:330-9.
15. Canadian Treatment Action Council. Generic drugs in Canada: A policy paper. World Health Stratigies, 2007.
16. Guidelines for the economic evaluation of health technologies: Canada. Ottawa: Canadian Agency for Drugs and Technologies in Health, 2006.
17. Paris V, Docteur E. Pharmaceutical pricing and reimbursement policies in Canada. Organisation for Economic Co-operation and Development, 2006.
18. Procedure for common drug review. Ottawa: Canadian Agency for Drugs and Technologies in health, 2010.
19. Demers V, Mela M, Jackevicius C, Cox J, Kalavrouziotois D, Rinfret S, et al. Comparison of provincial prescription drug plans and the impact on patients' annual drug expenditures. CMAJ. 2008;178:405-9.
20. Daw J, Morgan S. Stitching the gaps in the Canadian public drug coverage patchwork? A review of provincial pharmacare policy changes from 2000 to 2010. Health Policy. 2012;104:19-26.
21. Menon D, Stafinski T. Health technology assessment in Canada: 20 years strong? Value in Health. 2009;12:S14-9.
22. Bryndova L, Pavlokova K, Roubal T, Rokosova M, Gaskins. Czech Republic: Health System Review. European Observatory on Health Systems and Policies, 2009.
23. Davidova J, Praznovcova L, Lundborg C. Pricing and reimbursement of pharmaceuticals in the Czech Republic and Sweden. Pharm World Sci. 2008;30:57-64.
24. Strandberg-Larsen M, Nielsen M, Vallgarda S, Krasnik A, Vrangbaek K. Denmark: Health System Review. European Observatory on Health Systems and Policies, 2007.

25. Thomsen E, Er S, Rasmussen P. Pharmaceutical Pricing and Reimbursement Information: Denmark. Vienna: Gesundheit Osterreich GmbH / Geschaftsbereich OBIG (GOG/OBIG), 2008.
26. National Institute for Health and Clinical Excellence. Guide to the methods of technology appraisal. 2008.
27. Palnoch D, Kullman D, Sherriff J, Barnor E, Baxter M, Carter J, et al. Pharmaceutical Pricing and Reimbursement Information: United Kingdom. Vienna: Gesundheit Osterreich GmbH / Geschaftsbereich OBIG (GOG/OBIG), 2007.
28. Rawlins M, Barnett D, Stevens A. Pharmacoeconomics: NICE's approach to decision-making. Br J Clin Pharmacol. 2010;70:346-9.
29. Chalkidou K. Comparative effectiveness review within the U.K.'s National Institute for Health and Clinical Excellence. Issue Brief (Commonw Fund). 2009;59:1-12.
30. Pudersell K, Vetka A, Rootslane L, Mathiesen M, Vendla K, Laasalu J. Pharmaceutical Pricing and Reimbursement Information: Estonia. Vienna: Gesundheit Osterreich GmbH / Geschaftsbereich OBIG (GOG/OBIG), 2007.
31. Kanavos P, Vandoros S, Habicht J, de Johncheere K. Review of the Estonian pharmaceutical sector: Towards the development of a national medicines policy. Copenhagen: World Health Organization, 2009.
32. Finnish statistics on medicines 2010. Helsinki: Finnish Medicines Agency and Social Insurance Institution, 2011.
33. Peura S, Rajaniemi S, Kurkijarvi U. Pharmaceutical Pricing and Reimbursement Information: Finland. Vienna: Gesundheit Osterreich GmbH / Geschaftsbereich OBIG (GOG/OBIG), 2007.
34. Vuorenkoski L. Finland: Health System Review. European Observatory on Health Systems and Policies, 2008.
35. Van Ganse E, Chamba G, Bruet G, Becquart V, Stamm C, Lopes S, et al. Pharmaceutical Pricing and Reimbursement Information: France. Vienna: Gesundheit Osterreich GmbH / Geschaftsbereich OBIG (GOG/OBIG), 2008.
36. Sermet C, Andrieu V, Godman B, Van Ganse E, Haycox A, Reynier J. Ongoing pharmaceutical reforms in France. Appl Health Econ Health Policy. 2010;8:7-24.
37. Rochaix L, Xerri B. National Authority for Health: France. Issue Brief (Commonw Fund). 2009;58:1-9.
38. Nasser M, Sawicki P. Institute for Quality and Efficiency in Health Care: Germany. Issue Brief (Commonw Fund). 2009;57:1-12.
39. von der Schulenberg J, Prenzler A, Schurer W. Cancer management and reimbursement aspects in Germany: an overview demonstrated by the case of colorectal cancer. Eur J Health Econ. 2010;10:S21-6.
40. Stargardt T, Busse R. Pharmaceutical Pricing and Reimbursement Information: Germany. Vienna: Gesundheit Osterreich GmbH / Geschaftsbereich OBIG (GOG/OBIG), 2008.
41. Bekkering G, Kleijnen J. Procedures and methods of benefit assessments for medicines in Germany. Eur J Health Econ. 2008;9:S5-29.
42. Fricke F, Dauben H. Health technology assessment: A perspective from Germany. Value in Health. 2009;12:S20-7.
43. Vardica A, Kontozamanis V. Pharmaceutical Pricing and Reimbursement Information: Greece. Vienna: Gesundheit Osterreich GmbH / Geschaftsbereich OBIG (GOG/OBIG), 2007.
44. Yfantopoulos J. Pharmaceutical pricing and reimbursement reforms in Greece. Eur J Health Econ. 2008;9:87-97.
45. Kovacs T, Rozsa P, Szigeti S, Borcsek B, Lengyel G. Pharmaceutical Pricing and Reimbursement Information: Hungary. Vienna: Gesundheit Osterreich GmbH / Geschaftsbereich OBIG (GOG/OBIG), 2007.
46. Dasbach E, Nagy L, Brandtmuller A, Elbasha E. The cost effectiveness of a quadrivalent human papillomavirus vaccine (6/11/6/18) in Hungary. Journal of Medical Economics. 2010;13:110-8.
47. Halldorsson M. Health care systems in transition: Iceland. Copenhagen: 2003.
48. Barry M. Economics in drug usage in the Irish healthcare setting. 2008.
49. McDaid D, Wiley M, Maresso A, Mossialos E. Ireland: Health system review. Health Systems in Transition. 2009;11(4):1-268.
50. Tilson L, Barry M. Recent developments in pharmacoeconomics evaluation in Ireland. Expert Review of Pharmacoeconomics and Outcomes Research. 2010;10:221-4.

51. Elliott D, Byrne G. Pharmaceutical Pricing and Reimbursement Information: Ireland. Vienna: Gesundheit Osterreich GmbH / Geschaftsbereich OBIG (GOG/OBIG), 2007.
52. Barry M, Usher C, Tilson L. Public drug expenditure in the Republic of Ireland. Expert Rev Pharmacoeconomics Outcomes Res. 2010;10:239-45.
53. Barry M, Tilson L. Recent developments in pricing and reimbursement of medicines in Ireland. Expert Rev Pharmacoeconomics Outcomes Res. 2007;7:605-11.
54. Shani S, Siebzehner M, Luxenburg O, Shemer J. Setting priorities for the adoption of health technologies on a national level -- the Israeli experience. Health Policy. 2000;54:169-85.
55. Greenberg D, Siebzehner M, Pliskin J. The process of updating the National List of Health Services in Israel: Is it legitimate? Is it fair. International Journal of Technology Assessment in Health Care. 2009;25:255-61.
56. Folino-Gallo P, Montilla S, Bruzzone M, Martini N. Pricing and reimbursement of pharmaceuticals in Italy. Eur J Health Econ. 2008;9:305-10.
57. Lo Scalzo A, Donatini A, Orzella L, Cicchetti A, Profili S, Maresso A. Italy: Health system review. 2009.
58. Martini N, Folino-Gallo P, Montilla S. Pharmaceutical Pricing and Reimbursement Information: Italy. Vienna: Gesundheit Osterreich GmbH / Geschaftsbereich OBIG (GOG/OBIG), 2007.
59. Liu G, Fukuda T, Lee C, Chen V, Zheng Q, Kamae I. Evidence-based decision-making on medical technologies in China, Japan, and Singapore. Value in Health. 2009;12:S12-7.
60. Tatara K, Okamoto E. Japan: Health system review. Health Systems in Transition. 2009;11(5):1-164.
61. Iizuka T. The economics of pharmaceutical pricing and physician prescribing in Japan: Aoyama Gakuin University; 2008.
62. Nakao S, Kawabe H, Takuma H, Shiragami M. Acceptable incremental cost effectiveness ratio for use of new drugs, case in statin therapies. Yakugaku Zasshi. 2010;130:1347-52.
63. Kamae I. Value-based approaches to healthcare systems and pharmacoeconomics requirements in Asia. South Korea, Taiwan, Thailand and Japan. Pharmacoeconomics. 2010;28:831-8.
64. Habl C, Antony K, Arts D, Entleitner M, Froschl B, Leopold C, et al. Surveying, assessing and analysing the pharmaceutical sector in the 25 EU member states. Luxembourg: European Commission, 2006.
65. Caisse Nationale de la Sante. Formulaires d'inscription dans la liste positive 2011 [October 10, 2012]. Available from: http://www.cns.lu/prestataires/?m=55-41-54&p=46.
66. Oortwijn W, Mathijissen J, Banta D. The role of health technology assessment in selected middle-income countries. Health Policy. 2010;95:174-84.
67. Moise P, Docteur E. Pharmaceutical pricing and reimbursement policies in Mexcio. Paris: OECD, 2007.
68. Schafer W, Kroneman M, Boerma W, van den Berg M, Westert G, Deville W, et al. The Netherlands: Health system review. Health Systems in Transition. 2010;12(1):1-229.
69. Raftery J. Paying for costly pharmaceuticals: regulation of new drugs in Australia, England and New Zealand. Med J Aus. 2008;188:26-8.
70. Cumming J, Mays N, Daube J. How New Zealand has contained expenditure on drugs. BMJ. 2010;340:c2441.
71. Sundakov A, Sundakov V. New Zealand pharmaceutical policies: Time to take a fresh look. Castalia Strategic Advisors, 2005.
72. Festoy H, Sveen K, Yu L-M, Gjonnes L, Gregersen T. Pharmaceutical Pricing and Reimbursement Information: Norway. Vienna: Gesundheit Osterreich GmbH / Geschaftsbereich OBIG (GOG/OBIG), 2008.
73. Kuszewski K, Gericke C. Health systems in transition: Poland. Copenhagen: WHO Regional Office for Europe on behalf of the European Observatory on Health Systems and Policies, 2005.
74. Janiszewski R, Bondaryk K. Pharmaceutical Pricing and Reimbursement Information: Austria. Vienna: Gesundheit Osterreich GmbH / Geschaftsbereich OBIG (GOG/OBIG), 2007.
75. Kazakov R. Pricing and reimbursement policies in new EU accession countries. Journal of Generic Medicines. 2007;4:249-59.
76. Barros P, de Almeida Simoes J. Portugal: Health system review. Health Systems in Transition. 2007;9(5):1-140.

77. Teixeira I, Vieira I. Pharmaceutical Pricing and Reimbursement Information: Portugal. Vienna: Gesundheit Osterreich GmbH / Geschaftsbereich OBIG (GOG/OBIG), 2008.
78. Mazag J, Segec A. Pharmaceutical Pricing and Reimbursement Information: Slovakia. Vienna: Gesundheit Osterreich GmbH / Geschaftsbereich OBIG (GOG/OBIG), 2007.
79. Kalo Z, Paris V, Docteur E. Pharmaceutical pricing and reimbursement policies in Slovakia. Organisation for Economic Co-operation and Development, 2008.
80. Albreht T, Turk E, Toth M, Ceglar J, Marn S, Pribakovic Brinovec R, et al. Slovenia: Health system review. Health Systems in Transition. 2009;11(3):1-168.
81. Jirawattanapisal T, Kingkaew P, Lee T, Yang M. Evidence-based decision-making in Asia-Pacific with rapidly changing health-care systems: Thailand, South Korea, and Taiwan. Value in Health. 2009;12:S4-11.
82. Bae E, Lee E. Pharmacoeconomic guidelines and theier implementation in the positive list system in South Korea. Value in Health. 2009;12:S36-41.
83. Chun C, Kim S, Lee J, Lee S. Republic of Korea: Health system review. Health Systems in Transition. 2009;11(7):1-184.
84. Yang B-M, Bae E, Kim J. Economic evaluation and pharmaceutical reimbursement reform in South Korea's National Health Insurance. Health Affairs. 2008;27:179-87.
85. Park S, Lim S, Choi H, Lee S, Kim D, Yim E, et al. Evaluation on the first 2 years of the positive list system in South Korea. Health Policy. 2012;104:32-9.
86. Ngorsuraches S, Meng W, Kim B, Kulsomboon V. Drug reimbursement decision-making in Thailand, China, and South Korea. Value in Health. 2012;15:S120-5.
87. Duran A, Lara J, van Waveren M. Spain: Health system review. Health Systems in Transition. 2006;8(4):1-208.
88. Vogler S, Espin J, Habl C. Pharmaceutical pricing and reimbursement information (PPRI) - new PPRI analysis including Spain. Pharmaceuticals Policy and Law. 2009;11:213-34.
89. Dormuth C, Glynn R, Neumann P, Maclure M, Brookhart A, Schneeweiss S. Impact of two seequential drug cost-sharing policies on the use of inhaled medications in older patients with chronic obstructive pulmonary disease or asthma. Clin Ther. 2006;28:964-78.
90. Anell A, Persson U. Reimbursement and clinical guidance for pharmaceuticals in Sweden. Eur J Health Econ. 2005;50:274-9.
91. Glenngard A, Hjalte F, Svensson M, Anell A, Bankauskaite V. Health systems in transition: Sweden. Copenhagen: WHO Regional Office for Europe on behalf of the European Observatory on Health Systems and Policies, 2005.
92. Moise P, Docteur E. Pharmaceutical pricing and reimbursement policies in Sweden. Paris: OECD, 2007.
93. Pharmaceutical Benefits Board L. The Swedish pharmaceutical reimbursement system. 2007.
94. Redman T, Hoggard M. Pharmaceutical Pricing and Reimbursement Information: Sweden. Vienna: Gesundheit Osterreich GmbH / Geschaftsbereich OBIG (GOG/OBIG), 2007.
95. Paris V, Docteur E. Pharmaceutical pricing and reimbursement policies in Switzerland. Paris: OECD, 2007.
96. Decollogny A, Eggli Y, Halfon P, Lufkin. Determinants of generic substitution in Switzerland. BMC Health Services Research. 2011;11(17).
97. Tatar M. Pharmaceutical Pricing and Reimbursement Information: Turkey. Vienna: Gesundheit Osterreich GmbH / Geschaftsbereich OBIG (GOG/OBIG), 2007.
98. Balfour D, Evans S, Januska J, Lee H, Lewis S, Nolan S, et al. Medicare Part D - A roundtable discussion of current issues and trends. Journal of Managed Care Pharmacy. 2009;15:S3-9.
